# Prevalence of overweight and obesity in a South Texas cystic fibrosis center

**DOI:** 10.1016/j.jcte.2026.100445

**Published:** 2026-05-10

**Authors:** Radhika Pillai, Hussein Zaitoon, Wouter Koek, Kay Vavrina, Eric Ortiz, Maria Rayas

**Affiliations:** a Division of Pediatric Endocrinology and Diabetes, University of Texas Health Science Center San Antonio, 7703 Floyd Curl Drive, San Antonio, TX 78229, United States; b Long School of Medicine, University of Texas Health Science Center San Antonio, 7703 Floyd Curl Drive, San Antonio, TX 78229, United States; c University Health, 4502 Medical Drive, San Antonio, TX 78229, United States

**Keywords:** Cystic fibrosis, Obesity, Overweight, Ethnicity, Hispanic, Pulmonary function, Modulators

## Abstract

**Background:**

Overweight and obesity rates have increased drastically among people with cystic fibrosis (CF), in part due to advancements in care, including highly effective modulator therapies (HEMT). Ethnic differences in the prevalence of overweight/obesity in CF are unknown.

**Methods:**

To determine the prevalence of overweight/obesity by ethnicity, we performed a cross-sectional retrospective chart review of patients ≥ 2yrs old with CF at the University of Texas Health San Antonio CF Center. Logistic regression stratified by age group (children: 2–17 yr, adults: ≥18 yr) was used to evaluate factors associated with overweight/obesity among Hispanics and non-Hispanic whites after adjustment for confounders.

**Results:**

One hundred seventy-two patients (68 children/104 adults, 49% Hispanic, 47% female) were assessed. Overweight/obesity was prevalent in 35% of children and 42% of adults. Multivariable analysis revealed that the odds of overweight/obesity were lower in children (p = 0.032) and adults (p = 0.007) who used supplemental feeds, but higher among adults using HEMT (p = 0.037) and adults with higher FEV1pp (p = 0.016). Ethnic differences in overweight/obesity prevalence were not apparent in either age group.

**Conclusion:**

The odds of overweight/obesity were lower among children and adults using supplemental feeds, whereas HEMT use and having higher lung function were associated with increased odds among adults. Multi-center studies performed in diverse CF populations are essential to understand factors associated with overweight/obesity.

## Highlights

• In our diverse CF center, 35% of children and 42% of adults are overweight/obese.
• Modulator use and higher FEV1pp are linked to overweight/obesity in adults.
• Odds of overweight/obesity are lower with use of supplemental feeds.
• No ethnic differences in overweight/obesity were observed.

## Background

Cystic fibrosis (CF) is caused by genetic mutations in the CF transmembrane conductance regulator (CFTR) protein that impairs the function of multiple organs, including the lungs, pancreas, and gut [1]. Historically, CF has been characterized by undernutrition, partly due to pancreatic insufficiency and CF-related diabetes with insulin deficiency, as well as progressive pulmonary decline, and early death [1], [2]. In the past decade, however, significant advancements in CF care, including the availability of highly effective modulator therapies (HEMT), have improved nutrition, lung function, and life expectancy [1].

HEMT refers to CFTR modulators associated with substantial restoration of CFTR function and significant clinical benefit, most notably ivacaftor approved in 2012 and elexacaftor/tezacaftor/ivacaftor (ETI) approved in 2019 in eligible individuals [3], [4]. ETI has demonstrated efficacy in people who have at least one F508del mutation in the CFTR gene, dramatically increasing eligibility for treatment to a large majority of people with CF [5]. HEMT use has contributed to improved nutrition and weight gain through multifactorial mechanisms, including reduced energy expenditure from decreased inflammation, improved pancreatic sufficiency and intestinal absorption, and favorable changes in bile acid metabolism and the gut microbiome [6].

One unintended consequence of advancements in CF care has been a noticeable rise in overweight and obesity [7], [8], [9], with increasing prevalence observed even prior to widespread eligibility for HEMT [9]. An analysis of data from the CF Foundation Patient Registry collected between 2000 and 2019 showed that the prevalence of overweight and obesity doubled in the past 20 years, from 10% (8% overweight, 2% obese) to 24% (17% overweight, 7% obese) [9]. This poses a unique challenge, as historically there has been a strong emphasis on optimization of nutrition and weight gain to reduce risk of pulmonary impairment and mortality [1].

While the risk of developing overweight/obesity and associated complications is increased in Hispanics compared to non-Hispanic whites (NHW) in the general population [10], ethnic differences in the prevalence of overweight/obesity and associated risk factors in CF are unknown.

Our South Texas CF Center serves a population largely underrepresented in CF research, with approximately 50% of patients with CF self-identifying as Hispanic, which is significantly higher than the national average of 10.3% [11], [12]. This study aimed to assess the prevalence of overweight and obesity among Hispanics compared to NHW at the University of Texas Health San Antonio (UTHSA) CF Center. Understanding the prevalence of obesity in an ethnically diverse CF center can inform future screening practices and the development of individualized interventions to address obesity and its associated comorbidities.

## Methods

This was a retrospective cross-sectional chart review of patients with CF who received care at the UTHSA CF Center between January 2022 and January 2023. The study was reviewed and approved by the Institutional Review Board.

Patients with confirmed CF by sweat chloride testing or genetic analysis were included in the study if they were 2 years of age or older. Exclusion criteria included pregnancy within the 12 months of the study period, as this would confound the interpretation of body mass index (BMI) measurements.

Demographics (age, ethnicity, sex, insurance, annual household income), body mass index (BMI), CF-related variables (CF genotype classification, pancreatic function, supplemental feeding use, lung function, modulator therapy use), and cardiovascular risk factors (family history of diabetes, hemoglobin A1c, blood pressure, CFRD diagnosis) were extracted from the electronic health record. Blood pressure percentiles were collected for pediatric patients aged 17 and younger. BMI reported corresponds to the first value recorded during the study window. All other variables were assessed contemporaneously with this BMI measurement, except for lung function, which is reported as the peak forced expiratory volume in 1 s (FEV1pp) documented during the study period.

BMI was calculated as body weight (kg)/height (m^2^) and categorized using CDC pediatric criteria for BMI percentiles (age and sex specific) and WHO adult criteria, where adequate BMI is defined as 5–84%ile for pediatric patients, BMI 18.5–––24.9 kg/m^2^ for adult patients; and overweight/obese BMI ≥ 85%ile for pediatric patients, BMI ≥ 25 kg/m^2^ for adults patients.

Highly effective modulator therapy (HEMT) was defined as treatment with elexacaftor/tezacaftor/ivacaftor (ETI) or ivacaftor [3]. Patients treated with lumacaftor/ivacaftor (2 pediatric) and tezacaftor/ivacaftor (1 pediatric, 1 adult) were categorized in the ‘no HEMT’ group. Pancreatic sufficiency status was determined based on the need for pancreatic enzyme replacement therapy. Supplemental feeding use included oral supplements such as high-calorie shakes and/or gastrostomy tube feeding. Lung function was assessed as the peak FEV1pp reported during the study period.

CFTR genotypes were categorized as severe or mild based on the six established CFTR mutation classes, which reflect each mutation's functional impact. This classification approach is consistent with prior studies [13], [14].

A total of 181 patients met the study eligibility criteria. Due to the small number of patients in the underweight BMI category (n = 9; 2 pediatric and 7 adults), this group was excluded from final statistical analyses. They were not combined with the adequate BMI group as people that are underweight may represent a clinically distinct population when compared with patients of adequate BMI.

### Statistical analysis

Descriptive statistics were calculated for demographic and clinical characteristics and stratified by age group to correspond with the blood pressure percentiles obtained for pediatric patients (children: 2–17 years, adults: ≥18 years) and by BMI (*adequate*: pediatric patients 5–84%ile, adult patients BMI 18.5–––24.9 ; *overweight/obese*: pediatric patients ≥ 85%ile, adult patients BMI ≥ 25) . Pediatric and adult cohorts were assessed separately to account for anticipated differences in factors associated with overweight/obesity.

Continuous variables were expressed as mean +/- standard deviation (SD) and categorical variables as frequencies and percentages. Differences in group characteristics based on BMI (adequate, overweight/obese) were assessed using univariable logistic regression to aid in selecting the characteristics for inclusion in the multivariable analysis.

Multivariable logistic regression conducted separately in each age group (children: 2–17 years, adults: ≥18 years) was used to evaluate factors associated with overweight/obesity using the following independent variables: ethnicity, age, sex, and supplemental feeding. Exploratory predictors with a p-value of ≤ 0.2 in the univariable analyses were also included in the multivariable regression models. Primary multivariable analyses were conducted using multiple imputation to account for missing data (number of multiple imputations: 20; all variables used in the multivariable analyses were included as predictors). To assess the robustness of the findings, complete-case analyses were also performed. Statistical analyses were conducted using jamovi (version 2.7.15; https://www.jamovi.org), the R package “mice” (version 3.19.0) to conduct multiple imputation assuming a missing at random mechanism, and the R package “car” (version 3.1–5) to detect multicollinearity (i.e., high correlation among independent variables) by assessing variance inflation factors (values lower than 5 are generally considered acceptable). A significance level of p < 0.05 was used for all tests.

## Results

### Pediatric data

Sixty-eight children with CF (46% Hispanic, 51% female, 72% on HEMT) were assessed. Twenty-four children (35%) were overweight/obese (Table 1).

**Table 1:** Characteristics of Pediatric Patients with Cystic Fibrosis by Body Mass Index Category.

Characteristic	Overall N = 68 (100)	Normal BMI N = 44 (65)	Overweight/Obesity N = 24 (35)	p-value
Age (years)	10.44 (4.43)	10.89 (4.46)	9.63 (4.34)	0.261
Ethnicity				
Non-Hispanic	37/68 (54%)	24/44 (55%)	13/24 (54%)	0.976
Hispanic	31/68 (46%)	20/44 (45%)	11/24 (46%)	
Sex				
Female	35/68 (51%)	25/44 (57%)	10/24 (42%)	0.235
Male	33/68 (49%)	19/44 (43%)	14/24 (58%)	
Mutation classification*				
Severe	57/65 (88%)	40/42 (95%)	17/23 (74%)	0.024
Mild	8/65 (12%)	2/42 (4.8%)	6/23 (26%)	
(Missing)	3/68 (4%)	2/44 (5%)	1/24 (4%)	
HEMT use	49/68 (72%)	33/44 (75%)	16/24 (67%)	0.466
Pancreatic sufficiency	10/68 (15%)	2/44 (4.5%)	8/24 (33%)	0.005
Supplemental feeding	34/68 (50%)	28/44 (64%)	6/24 (25%)	0.003
HbA1c (%)^†^	5.63 (0.66)	5.75 (0.74)	5.39 (0.37)	0.055
(Not obtained)	16/68 (24%)	9/44 (20%)	7/24 (29%)	
CFRD	4/68 (5.9%)	4/44 (9.1%)	0/24 (0%)	0.993
FEV₁ (% predicted)^‡^	100.28 (14.94)	99.05 (15.83)	102.41 (13.34)	0.401
(Missing)	8/68 (12%)	6/44 (14%)	2/24 (8%)	
Systolic blood pressure (%)	81.28 (18.51)	78.39 (20.18)	86.58 (13.83)	0.089
Diastolic blood pressure (%)	79.28 (18.85)	79.23 (18.91)	79.38 (19.13)	0.975
Public insurance^¶^	40/67 (60%)	28/44 (64%)	12/23 (52%)	0.365
(Missing)	1/68 (1%)	0/44 (0%)	1/24 (4%)	
Annual income				
> $30,000	45/68 (66%)	26/44 (59%)	19/24 (79%)	0.10
< $30,000	23/68 (34%)	18/44 (41%)	5/24 (21%)	
Family history of diabetes	32/68 (47%)	20/44 (45%)	12/24 (50%)	0.72

Data presented as n (%), mean ± SD; HEMT, highly effective modulator therapy, *n = 65, ^†^n = 52, ^‡^n = 60, ^¶^n = 67. P values obtained by univariable logistic regression.

When analyzed univariably, the odds of overweight/obesity in pediatric patients were lower with supplemental feeding use (P = 0.003) and higher with pancreatic sufficiency (P = 0.005) and milder CFTR genotype (P = 0.024, Table 1). Systolic blood pressure (BP) percentiles, annual household income above $30,000 (poverty threshold for a family of 4 in Texas), and hemoglobin A1c tended to be related to BMI (0.05 < P < 0.1, Table 1).

Although hemoglobin A1c had a p-value ≤ 0.2 in the univariable analysis, it was excluded from the multivariable model as it is not routinely obtained until approximately 10 years of age and was therefore unavailable for 24% of the pediatric cohort. Given that this missingness was structural rather than random, imputing hemoglobin A1c values would violate assumptions of multiple imputation and risk introducing bias.

**Fig. 1:**
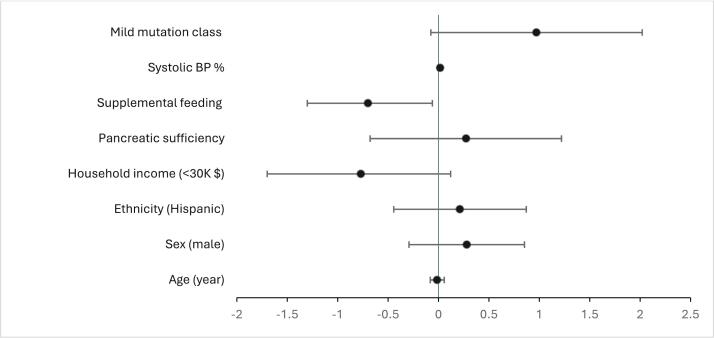
Forest plot of multivariable logistic regression with multiple imputation results among pediatric patients. Odds ratios (OR) and their 95% confidence intervals (CI) are shown on a logarithmic scale.

Multivariable analysis (variance inflation factors < 1.9; missing data at most 4% [Mutation classification]) revealed that the odds of overweight/obesity were lower with supplemental feeding use (OR 0.20, 95% CI 0.05–0.87, P = 0.032) (Supplemental Table 1 & Fig. 1). Milder mutation class (OR 9.38, 95% CI 0.84–105.0, p = 0.069) showed a non-significant trend with overweight/obesity. However, ethnic differences in overweight/obesity prevalence were not apparent (OR 1.63, 95% CI 0.36–7.43, p = 0.521). Multiple imputation and complete-case analyses yielded similar OR estimates and p-values for all variables (Supplemental Table 1).

#### Adult data

One hundred four adults with CF (51% Hispanic, 44% female, 83% on HEMT) were assessed. Forty-four adults (42%) were overweight/obese (22 Hispanic) (Table 2).

**Table 2:** Characteristics of Adult Patients with Cystic Fibrosis by Body Mass Index Category.

Characteristic	Overall N = 104 (100)	Normal BMI N = 60 (58)	Overweight/Obesity N = 44 (42)	p-value
Age (years)	29.77 (9.39)	29.20 (10.45)	30.55 (7.74)	0.470
Ethnicity*				
Non-Hispanic	50/103 (49%)	29/60 (48%)	21/43 (49%)	0.96
Hispanic	53/103 (51%)	31/60 (52%)	22/43 (51%)	
Sex				
Female	46/104 (44%)	30/60 (50%)	16/44 (36%)	0.168
Male	58/104 (56%)	30/60 (50%)	28/44 (64%)	
Mutation classification^†^				
Severe	82/97 (85%)	48/54 (89%)	34/43 (79%)	0.19
Mild	15/97 (15%)	6/54 (11%)	9/43 (21%)	
(Missing)	7/104 (7%)	6/60 (10%)	1/44 (2%)	
HEMT use	86/104 (83%)	46/60 (77%)	40/44 (91%)	0.067
Pancreatic sufficiency	15/104 (14%)	8/60 (13%)	7/44 (16%)	0.712
Supplemental feeding	22/104 (21%)	20/60 (33%)	2/44 (4.5%)	0.002
HbA1c (%)^‡^	6.20 (1.35)	6.11 (1.23)	6.33 (1.51)	0.421
(Missing)	5/104 (5%)	2/60 (3%)	3/44 (7%)	
CFRD	43/104 (41%)	23/60 (38%)	20/44 (45%)	0.467
FEV₁ (% predicted)*	75.94 (23.46)	70.24 (25.10)	83.59 (18.73)	0.006
• (Missing)	1/104 (1%)	1/60 (2%)	0/44 (0%)	
Systolic blood pressure (mmHg)	125.35 (12.25)	122.88 (11.79)	128.70 (12.19)	0.019
Diastolic blood pressure (mmHg)	80.14 (9.26)	78.73 (9.78)	82.07 (8.23)	0.071
Public insurance ^¶^	49/100 (49%)	25/57 (44%)	24/43 (56%)	0.238
(Missing)	4/104 (4%)	3/60 (5%)	1/44 (2%)	
Annual income < $30,000^§^	29/98 (30%)	18/56 (32%)	11/42 (26%)	0.523
(Missing)	6/104 (6%)	4/60 (7%)	2/44 (5%)	
Family history of diabetes	59/104 (57%)	30/60 (50%)	29/44 (66%)	0.108

Data presented as n (%), mean ± SD; HEMT, highly effective modulator therapy, *n = 103, ^†^n = 97, ^‡^n = 99, ^¶^n = 100, ^§^n = 98. P values obtained by univariable logistic regression.

Univariable analysis showed that the odds of overweight/obesity in adults were lower with supplemental feeding use (P = 0.002) and increased with higher FEV1pp (P = 0.006) and higher systolic blood pressure (P = 0.019, Table 2). Treatment with HEMT and diastolic blood pressure tended to be related to overweight/obesity (0.05 < P < 0.1, Table 2).

**Fig. 2:**
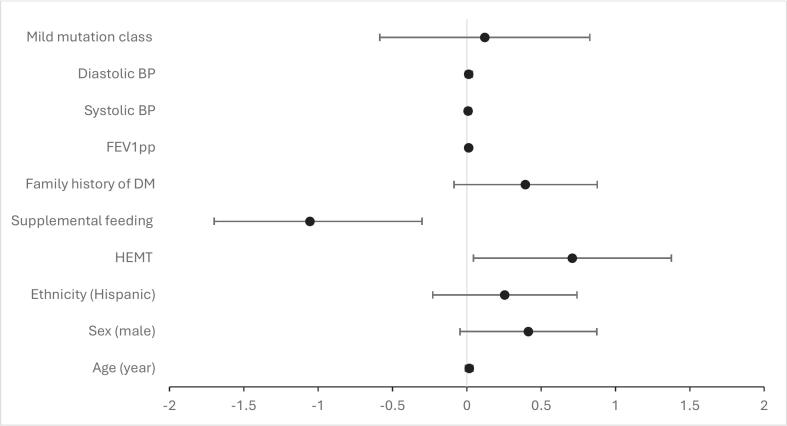
Forest plot of multivariable logistic regression with multiple imputation analysis among adult patients. Odds ratios (OR) are presented on the logarithmic scale with corresponding 95% confidence intervals (CI).

Multivariable analysis (variance inflation factors < 1.6; missing data at most 7% [Mutation classification]) revealed that the odds of overweight/obesity in adults was lower with supplemental feeding use (OR 0.088, 95% CI 0.02–0.50, P = 0.007) and increased with HEMT (OR 5.13, 95% CI 1.11–23.74, p = 0.037) and with higher FEV1pp (OR 1.03, 95% CI 1.01–1.06, P = 0.016) (Supplemental Table 2 & Fig. 2). Again, no ethnic differences were apparent (OR 1.80, 95% CI 0.59–5.51, p = 0.30). Multiple imputation and complete-case analyses yielded similar OR estimates and p-values, except for HEMT (p-value statistically significant and lower with multiple imputation than with complete-case analysis, Supplemental Table 2).

## Discussion

In this retrospective study, we observed a prevalence of overweight/obesity in 35% of children and 42% in adults at our CF center. Multivariable analysis revealed that the odds of overweight/obesity were lower in children and adults using supplemental feeds, but higher among adults with HEMT use and better lung function. Although we did not find ethnic differences, to our knowledge, this is the first study to examine the prevalence of overweight/obesity within a diverse pediatric and adult CF population following the widespread introduction of ETI therapy in 2019.

A rise in BMI has been appreciated in people with CF over time as advancements in care have improved nutritional status and pulmonary function. A cross-sectional study in pediatric patients at the Pittsburgh CF Center in 2012 found 23% of children were overweight/obese [15]. A single-center study conducted at the Minnesota Adult CF Center between 2015 and 2017 reported a combined prevalence of overweight and obesity of 32% among adults with CF [14]. More recently, data from the 2023 CF Foundation Patient Registry demonstrate a substantial increase in the proportion of adults with CF classified as overweight or obese, rising from 17% in 2003 to 41% in 2023 (28% overweight, 13% obese) [11]. These findings highlight a rising burden of excess weight that may increasingly parallel trends in the general U.S. adult population, where obesity was estimated at 40.3% based on 2022–2023 National Health and Nutrition Examination Survey data [16].

While no ethnic differences were observed, overweight and obesity prevalence across CF centers should be considered within regional demographic and epidemiologic contexts. Our South Texas CF center serves a large Hispanic population, with ∼ 30% of patients living below the poverty line (Table 1, Table 2). In Texas, youth obesity ranks sixth nationally, with the highest rates among Hispanic children, while adult obesity ranks 19th, with higher rates in South Texas [17], [18]. Beyond ethnicity, adverse social determinants of health associated with obesity—such as socioeconomic status (annual income < $20,000/year has been linked to obesity in CF), health care access, and food insecurity should be considered [9], [19]. These region-specific factors underscore the importance of incorporating community and social drivers, including dietary influences (e.g., sugar sweetened beverages, sodium intake, fast-food availability) and neighborhood characteristics (e.g., crime, green space, sidewalks), when evaluating obesity and related complications to inform more individualized CF care.

In children, multivariable analysis showed lower odds of overweight/obesity among youth using supplemental feeds. Supplemental feeding was selected as a variable of interest based on prior CF obesity studies [14], [20] and recent concerns that high-calorie intake practices may persist in the HEMT era [6]. Our findings suggest the CF team is appropriately reserving supplementation for patients requiring additional support to meet BMI goals established by the CF Foundation. Less severe CFTR genotypes showed a non-significant trend with overweight/obesity (p = 0.069). Although pancreatic sufficiency was linked to overweight/obesity in univariable analysis (p = 0.005), its correlation with less severe genotype may explain why this association was no longer significant after adjustment. We did not observe an association between overweight/obesity and HEMT use, which may reflect limited exposure, as only children ≥ 6 years were eligible for ETI during the study period. Characteristics of children with overweight/obesity cited in the literature include pancreatic sufficiency, less severe mutation class, and less use of supplemental feeds [20], [21], [22], [23]. While the long-term cardiovascular risk associated with overweight and obesity in children with CF is not well documented, childhood obesity in the general population is a well-established risk factor for atherosclerotic cardiovascular disease and is associated with approximately double the risk of dyslipidemia, hypertension, and type 2 diabetes [24]. As survival continues to improve with widespread use of HEMT, the prevalence of central features of metabolic syndrome (insulin resistance, visceral adiposity, and dyslipidemia) in children with CF may emerge.

In adults with CF, multivariable analysis also revealed overweight/obesity was less associated with use of supplemental feeds, whereas positive associations were observed with HEMT use and higher FEV1pp. HEMT use has been linked to increases in body fat, dyslipidemia, and hypertension in people with CF, suggesting a growing prevalence of metabolic syndrome in this population [25]. Ratti et al. observed that the number of adults with CF meeting criteria for metabolic syndrome more than doubled after one year of ETI therapy, increasing from 13 to 30 individuals [26]. In regards to higher FEV1pp, there is a well-established positive association between improved pulmonary function and BMI [1]. However, it is important to understand the temporal relationship between obesity and pulmonary function and whether there is a limit to the observed improvement in pulmonary function. Previous studies in CF have noted an upper BMI cut-off of 23, 25, and 28–29 kg/m2, respectively, above which improvement in pulmonary function was minimal [14], [27], [28]. In the general adult population, obesity is associated with negative health outcomes, including hypertension, hyperlipidemia, diabetes, cardiovascular disease, and more [16]. Although systolic blood pressure was associated with overweight and obesity in univariable analysis (p = 0.019), this association was no longer statistically significant after multivariable adjustment. It is not clear if overweight/obesity increases the risk of cardiovascular disease in adults with CF [29]. However, existing studies have demonstrated increased blood pressure, insulin resistance, and lipid profiles among adults with CF who are overweight/obese [7], [14], [22], [25].

As the life expectancy of people with CF continues to improve and the prevalence of obesity in CF continues to rise, understanding long-term clinical outcomes related to pulmonary function and cardiometabolic factors is warranted. Larger, prospective, longitudinal studies in regionally and ethnically diverse CF populations are needed to understand what factors are associated with greater predisposition of obesity in children and adults in the era of HEMT availability.

There are important limitations to our study. The cross-sectional design of the study limits our ability to infer causal and temporal relationships. This is of particular importance in understanding the impact of duration of HEMT on changes in weight and emergence of obesity-related complications in the pediatric and adult population. Our retrospective design also limited the availability of certain variables, such as lipid profiles, which were missing in many participants and prevented analysis. Univariable and multivariable logistic regression analyses are limited by potential residual confounding from unmeasured factors influencing weight in CF, including diet, physical activity, and HEMT treatment adherence. In addition, collinearity among CF-specific variables (e.g., pancreatic status and CFTR mutation classification) may have limited the precision of effect estimates (note, however, that the variance inflation factors obtained in the preset study (i.e., < 1.9) were all below those that generally raise concern about the reliability of the model’s estimates (i.e., 5–10). The dichotomization of BMI into adequate vs overweight/obesity may have reduced sensitivity to detect more nuanced associations across the weight spectrum. Finally, our study was conducted at a single center which may limit its generalizability.

## Conclusion

In our ethnically diverse CF center located in South Texas, overweight/obesity is prevalent in one third of the pediatric population and nearly half of adults. Multi-center, prospective, longitudinal studies performed in ethnically and regionally diverse CF populations are imperative to provide insights regarding screening and management of obesity and related comorbidities.

## CRediT authorship contribution statement

**Radhika Pillai:** Writing – original draft, Methodology, Investigation, Data curation, Conceptualization. **Hussein Zaitoon:** Writing – review & editing, Visualization. **Wouter Koek:** Writing – review & editing, Visualization, Validation, Formal analysis. **Kay Vavrina:** Writing – review & editing, Visualization, Data curation. **Eric Ortiz:** Writing – review & editing, Data curation. **Maria Rayas:** Writing – review & editing, Supervision, Methodology, Investigation, Funding acquisition, Data curation, Conceptualization.

## Declaration of competing interest

The authors declare that they have no known competing financial interests or personal relationships that could have appeared to influence the work reported in this paper.

## Data Availability

The data supporting this study are available from the corresponding author upon reasonable request.
