# Supplementary material for: Prevalence of overweight and obesity in a South Texas cystic fibrosis center
Source: J Clin Transl Endocrinol. 2026 May 10;45:100445. doi: 10.1016/j.jcte.2026.100445 (PMC13199930; doi:10.1016/j.jcte.2026.100445)
Supplement: Supplementary Data 1 [file mmc1.docx]

Supplementary Tables

Supplementary Table 1. Multivariable logistic regression for overweight/obesity in children

|  | **Multiple imputation (n=68)** | | **Complete-case analysis (n=65)** | |
| --- | --- | --- | --- | --- |
| **Variable** | **OR (95% CI)** | **p-value** | **OR (95% CI)** | **p-value** |
| Age (year) | 0.97 (0.83–1.14) | 0.708 | 0.97 (0.83-1.13) | 0.684 |
| Sex (male) | 1.91 (0.51–7.13) | 0.330 | 1.81 (0.5-6.62) | 0.368 |
| Ethnicity (Hispanic) | 1.63 (0.36–7.43) | 0.521 | 1.59 (0.37-6.94) | 0.535 |
| Household income (<30K $) | 0.17 (0.02–1.32) | 0.089 | 0.16 (0.02-1.24) | 0.079 |
| Pancreatic sufficiency | 1.88 (0.21–16.6) | 0.562 | 1.67 (0.2-13.99) | 0.637 |
| Supplemental feeding | 0.20 (0.05–0.87) | **0.032** | 0.22 (0.05-0.91) | **0.036** |
| Systolic BP | 1.04 (0.99–1.08) | 0.121 | 1.03 (0.99-1.08) | 0.127 |
| Mild mutation class | 9.38 (0.84–105.0) | 0.069 | 9.25 (0.87-98.22) | 0.065 |

Bold text indicates statistically significant associations (*p* < 0.05).
Abbreviations: OR, odds ratio; CI, confidence interval; BP, blood pressure.

Supplementary Table 2. Multivariable logistic regression for overweight/obesity in adults

|  | **Multiple imputation (n=104)** | | **Complete-case analysis (n=95)** | |
| --- | --- | --- | --- | --- |
| **Variable** | **OR (95% CI)** | **p-value** | **OR (95% CI)** | **p-value** |
| Age (year) | 1.04 (0.98–1.10) | 0.212 | 1.05 (0.97-1.12) | 0.224 |
| Sex (male) | 2.59 (0.90–7.50) | 0.078 | 3.07 (1.05-9.01) | **0.041** |
| Ethnicity (Hispanic) | 1.80 (0.59–5.51) | 0.30 | 1.88 (0.6-5.91) | 0.280 |
| HEMT | 5.13 (1.11–23.74) | **0.037** | 2.35 (0.48-11.39) | 0.291 |
| Supplemental feeding | 0.088 (0.02–0.50) | **0.007** | 0.09 (0.02-0.53) | **0.008** |
| Family history of DM | 2.48 (0.82–7.54) | 0.107 | 2.21 (0.72-6.84) | 0.168 |
| FEV1pp | 1.03 (1.01–1.06) | **0.016** | 1.03 (1.0-1.06) | **0.021** |
| Systolic BP | 1.02 (0.98–1.07) | 0.292 | 1.04 (0.99-1.09) | 0.146 |
| Diastolic BP | 1.03 (0.98–1.09) | 0.249 | 1.03 (0.97-1.09) | 0.381 |
| Mild mutation class | 1.32 (0.26–6.72) | 0.737 | 1.45 (0.29-7.14) | 0.649 |

Bold text indicates statistically significant associations (*p* < 0.05).
Abbreviations: OR, odds ratio; CI, confidence interval; HEMT, highly effective modulator therapies; BP, blood pressure; DM, diabetes mellitus; FEV1pp, forced expiratory volume in 1 second percent predicted.
